# Day-to-day associations between testosterone, sexual desire and courtship efforts in young men

**DOI:** 10.1098/rspb.2024.1508

**Published:** 2024-11-27

**Authors:** Tikal Catena, Blair T. Crewther, Adar B. Eisenbruch, Rachel L. Grillot, Dario Maestripieri, James R. Roney

**Affiliations:** ^1^ Department of Psychological and Brain Sciences, University of California, Santa Barbara, CA, USA; ^2^ Institute of Sport, National Research Institute, Warsaw, Poland; ^3^ School of Science and Technology, University of New England, Armidale, Australia; ^4^ Department of Psychology, Purchase College, State University of New York, Purchase, NY, USA; ^5^ Department of Comparative Human Development, The University of Chicago, Chicago, IL, USA

**Keywords:** testosterone, sexual desire, courtship, human mating

## Abstract

Testosterone plays important roles in reproductive behaviour in many species. Despite a common belief that testosterone regulates fluctuations in human sexual desire, there is little direct evidence that relates within-person changes in natural testosterone production to within-person changes in sexual desire. Here, we measured daily salivary testosterone concentrations from 41 adult men for one month, along with daily self-reports of sexual desire (*n* = 759 observations for the main analyses). We analysed concurrent relationships between within-person changes in testosterone and desire, and also lagged relationships that were analysed using a continuous-time modelling framework. We found no evidence for significant, positive relationships between testosterone and desire, which argues against the notion that day-to-day changes in eugonadal men’s baseline testosterone regulates changes in their sexual desire. However, additional analyses provided preliminary evidence for a positive relationship between testosterone and self-reported courtship effort, particularly on days when single participants interacted with potential romantic partners. Our findings add original evidence regarding day-to-day associations between testosterone and desire, and suggest that testosterone above minimum threshold concentrations does not increase sexual desire. We propose that the evolved functions of testosterone in human males are more closely associated with courtship efforts than with sexual desire.

## 1. Introduction

Do fluctuations in men’s baseline testosterone concentrations predict changes in their sexual desire? Statements by researchers such as ‘Testosterone is the major libidinal enhancer in both women and men’ ([1], p. 8) promote what appears to be a widespread belief in a strong relationship between these variables. Differential testosterone concentrations have also been theorized to explain variation in desire between men, as well as sex differences in sexual desire (e.g. [2–4]). A likely consequence of this assumption is that testosterone is commonly marketed and prescribed as a treatment for low sexual desire, even among patients lacking a formal diagnosis of hypogonadism [5]. What is the empirical evidence for these common beliefs regarding the crucial role of testosterone in regulating men’s sexual desire? The present research will focus on within-subject relationships between testosterone and desire: as testosterone increases and decreases from day to day within individuals, are there corresponding changes in sexual desire?

The question of within-individual relationships between hormones and sexual desire has been investigated more extensively in women than in men. Daily diary studies have provided evidence that within-cycle fluctuations in salivary estradiol concentrations positively predict shifts in women’s sexual desire, whereas fluctuations in progesterone are negative predictors of day-to-day changes in desire [6,7] (see also [8]; cf. [9]). However, as reviewed below, studies on male sexuality with equivalent designs are scarce and provide very limited evidence regarding day-to-day associations between men’s hormones and their sexual desire. This study aims to help fill this gap in the human sexuality literature.

### (a) Prior literature on relationships between testosterone and men’s sexuality

Prior research on the relationship between natural testosterone production and sexual desire has produced primarily mixed or non-significant findings. The majority of studies have used between-subject designs, addressing the question of whether men with higher baseline testosterone concentrations also experience higher sexual desire compared with men with lower testosterone. These studies have examined varied populations and measurement distributions, with two main types of comparisons: single hormone samples and their associated self-reported sexual desire [10–12] and equivalent comparisons based on averages of repeated measures (e.g. [13]). The emphases of the research designs also vary, including studies of age-related declines in testosterone and sexual function [14–16], or of couples’ hormone concentrations and dyadic sexual interactions [17]. Table 1 lists between-subjects studies examining associations between testosterone and sexual desire in male populations. Most studies report non-significant results, suggesting that the few significant correlations found may represent false positives or effects of unknown moderators. In an earlier review of this literature, van Anders [19] also concluded that the evidence for a positive relationship between testosterone and men’s desire was weak, and posited that common beliefs in stronger relationships may arise from stereotypes that associate both testosterone and desire with colloquial conceptions of masculinity.

**Table 1.** Between-subject studies of the relationship between testosterone and sexual desire. SDI, Sexual Desire Inventory [18].

citation	sexual desire measures	testosterone measure(s)	population	association type	association details	subject count
van Anders & Dunn [10]	SDI	single samples	adult males with mean age = 21	mixed	significant positive association between testosterone and the solitary desire component, but not dyadic or total desire	91
van Anders *et al*. [11]	SDI	single samples	adult males aged 19–54 (*M* = 31.5)	null	—	47
van Anders [12]	SDI	single samples	adult males (mean age = 23.3)	null	—	105
Schiavi *et al*. [16]	custom scale	averaged repeated measures from one night	older males aged 45–74	positive	testosterone significantly correlated with sexual desire	77
Mazur *et al*. [14]	custom scale	average of two samples, 1 h apart	older males aged 50–80	null	—	90
Sadowsky *et al*. [15]	custom scale	single samples	older males aged 65–80	null	—	60
Brown *et al*. [13]	custom scale	average of two samples, one week apart	adult males aged 20–30 (*M* = 22)	null	—	101

A second line of evidence comes from testosterone administration studies. It has been well established that chemical suppression of testosterone to hypogonadal concentrations can reduce men’s sexual desire, fantasy and frequency of sexual behaviour (e.g. [20,21]), but one study found that testosterone replacement at doses that were only half the average baseline concentrations were able to fully restore all sexual measures to their pre-suppression levels [20]. Conversely, the administration of supraphysiological doses of testosterone to eugonadal men had no effects on sexual desire [22,23]. Other research has shown that for hypogonadal men with low desire, testosterone administration that brings concentrations into the normal range can increase desire (e.g. [24]). Importantly, however, meta-analyses show that testosterone treatment is reliably effective only for men with relatively severe hypogonadism, whereas testosterone administration has no reliable effects for men with natural testosterone production in the normal range [25] (see also [26]). Together, these findings suggest that men’s sexual desire is suppressed when testosterone concentrations fall into the hypogonadal range, but that once concentrations are within the average range, desire does not reliably respond to further manipulations of testosterone doses.

The results of administration studies lead one to expect that day-to-day variability in testosterone and sexual desire will not be strongly associated in eugonadal men, since most of the hormone variability is expected to fall within the normal range of testosterone concentrations. Nonetheless, administration studies do not provide perfect evidence on this question, since they entail artificial suppression or supraphysiological administration of hormones, or the study of men with hormone concentrations outside the eugonadal range. Repeated, within-individual sampling of testosterone and desire would provide a more direct test of the relationship between natural testosterone production and sexual desire among eugonadal men.

Surprisingly few prior studies have provided data on within-subject relationships between men’s natural testosterone production and their sexual desire. A study by Stern *et al*. [27], in which men’s testosterone and desire were measured weekly for up to five weeks, may have been the most direct investigation of this question to date. In mixed regression models including simultaneous effects of testosterone, cortisol and their interactions, there were no significant within-subject predictors of desire. Desire was measured with the Sexual Desire Inventory (SDI) [18], however, which uses trait-like wording that may not be ideal for assessing state-like changes in desire. Rosenfield *et al*. [28] also used SDI scores and tested similar models, but with only two within-subject data points spaced about four months apart: they found no main effects of testosterone on desire, but a positive testosterone by cortisol interaction in predicting changes in men’s scores on the solitary desire subscale of the SDI. Raisanen *et al*. [29] measured hormones and the SDI in up to 12 monthly sessions and reported no main effects of within-subjects changes in testosterone on desire, though this was assessed in complex models that included cortisol, perceived stress and multiple control variables. Moreover, the same authors reported a testosterone by cortisol interaction in predicting men’s solitary desire only, but opposite in direction to the interaction reported by Rosenfield *et al*. [28]. Finally, Righetti *et al*. [9] did assess a state-like measure of desire for sexual activity daily across 15 days, but only tested associations with men’s testosterone within dyadic mixed regression models that also included their women partners’ ovarian hormones; they reported non-significant associations between testosterone and desire within these models.

In summary, no prior studies appear to have directly assessed the zero-order relationship between daily fluctuations in men’s natural testosterone and corresponding shifts in state-like measures of their sexual desire. The studies that have come closest to this have all reported non-significant relationships between testosterone and desire when considering the main effect of testosterone. An aim of the present study is to provide more definitive evidence on this question.

### (b) Testosterone and other components of mating effort

Functional accounts of testosterone stress its role in mate competition more than its possible role in promoting sexual desire. The ‘challenge hypothesis', for instance, posits that in seasonally breeding birds only a minimum breeding season baseline testosterone concentration is necessary for sexual behaviours, whereas the highest testosterone concentrations occur when males are addressing competitive challenges related to territory defence, mate attraction and mate guarding [30]. Other theoretical models emphasize that testosterone coordinates a broad suite of morphological (e.g. increased muscle mass), physiological (e.g. faster metabolic rate) and psychological effects (e.g. increased competitiveness and reactive aggression) that combined to promote more effective mate competition over the course of evolution [31–33].

Empirical patterns in human males are broadly consistent with these models. Testosterone tends to be elevated among single men relative to men in long-term relationships and those who are investing fathers (reviewed in [34–36]), which is consistent with a functional role for testosterone in mediating life history related tradeoffs between mating and parenting effort. Specific models have proposed temporal sequences in which elevated testosterone promotes enhanced mating effort that when successful (e.g. via relationship entry) leads to lowered testosterone and thus subsequently reduced mating effort [37,38]. Central to these arguments is the idea that testosterone is positively related mate competition efforts.

These models raise the possibility that transient shifts in testosterone are more related to courtship and mate competition than to felt sexual desire. Multiple studies have provided evidence for rapid increases in men’s salivary testosterone concentrations 15–45 min after brief social interactions with potential mates [39–42] (reviewed in [43]), and rapid testosterone elevations have in turn been associated with a suite of short-term effects that should promote more effective courtship and mate competition efforts (reviewed in [44]). These findings raise the possibility that day-to-day shifts in men’s testosterone may be positively correlated with corresponding shifts in their efforts at mate attraction. A secondary aim of this study is to assess preliminary evidence for this conjecture.

### (c) The present study

The primary aim of this study was to test the possible day-to-day association between men’s testosterone and their sexual desire. To this end, we collected daily measures of salivary testosterone for one month (31 days), as well as self-reports of sexual desire and other states or events relevant to mating effort on days corresponding to the hormone measures. This allowed us to test direct relationships between testosterone and sexual desire at the daily time scale, which appears to be novel in this literature. Based on the results of testosterone administration studies on eugonadal men reviewed above, we did not expect to find a significant within-subject relationship between testosterone and self-reports of sexual desire.

In addition to testing same-day associations between testosterone and desire, our density of data collection allowed tests of time-lagged associations between the two variables. Steroid hormones act in part via effects on gene transcription that can produce delayed effects [45]; conversely, hormone production might be affected by events or behaviours that occurred several days earlier [46]. We used continuous time (CT) modelling [47] to test time-lagged relationships between testosterone and desire: in other words, to test whether testosterone on a given day predicts sexual desire on subsequent days, and conversely, whether desire on a given day predicts testosterone on future days. See §2e for a further description of these models.

As a secondary aim, we tested whether testosterone was associated with daily self-reports of courtship efforts and interactions with potential mates. Based on the theorized relationship between testosterone and mating effort, we predicted a positive, within-subject, same-day association between testosterone and self-reports of effort put into mate attraction. Because mate attraction efforts are expected to vary by relationship status, we also examined the influence of relationship status on the association between hormones and mating effort, as well as on the association between hormones and sexual desire.

Although we were primarily interested in the relationship between testosterone and sexual desire, we also performed exploratory analyses involving cortisol, since cortisol assays were performed on the same saliva samples. Comprehensive analyses of the contemporaneous and lagged relationships between cortisol and testosterone in this sample were reported elsewhere [48]. The dual hormone hypothesis [49,50] predicts that some behavioural effects of testosterone may occur only when cortisol is low. Therefore, following prior studies in this literature [27–29], we also tested for testosterone by cortisol interactions in the prediction of courtship efforts and sexual desire.

## 2. Material and methods

### (a) Participants

Participants were 41 adult men aged 18 to 26 (*M* = 20.17, s.d. = 2.16) recruited from a subject pool at the University of California, Santa Barbara. Twenty-three participants self-reported their ethnicity as White, seven Hispanic, six Asian, three multiple ethnicities and two reported ‘other’. Participants were screened for a history or current use of anabolic steroids. Twenty-seven participants reported being single at the start of the study, while the remaining 14 were in a committed romantic relationship. Three participants reported a non-heterosexual sexual orientation (one self-reported being gay and two bisexual), but their exclusion did not affect any statistical conclusions and all participants were thus included in the final analyses. Participants were paid up to US$175 if they had no more than three missing sample days and participated in two in-person lab sessions (at which additional measures were collected that are not reported here), and were paid lower, pro-rated amounts if they had more missing data. All participants provided written, informed consent for their participation.

### (b) Procedures

After the informed consent process, participants provided demographic information (e.g. age, ethnicity, sexual orientation) and completed a battery of survey measures that are not analysed here (e.g. on personality and sociosexual orientation). For the next 31 days, participants then provided daily saliva samples and completed a daily online survey. On weekdays, the participants reported to a research lab to provide saliva samples in person. The target time for sample production was the early afternoon (mean = 13.30 h, s.d = 1:12 h) to minimize variance in hormone concentrations due to circadian fluctuations [51]. On weekend days, the men collected saliva samples at home and stored them in their own freezers until delivery at the next in-person lab visit. Saliva was collected via passive drool into polypropylene vials and samples were stored at −80°C until shipping for assay. Due to a data collection error, time of collection was not recorded for saliva samples collected on weekends, and thus the weekend samples were excluded in analyses that controlled for time of day. Participants completed the daily online surveys in the morning on a secure website and answered questions about events and experiences from the previous day.

### (c) Daily survey measures

The online survey queried participants about various behaviours, events and mood states across the prior day. Here, we analysed specific target variables related to sexual desire and courtship efforts. Sexual desire was assessed as a composite score from three questions: (i) ‘Yesterday, how much did you have sexual thoughts?’ (ii) ‘Yesterday, how much did you have sexual fantasies?’ (iii) ‘Yesterday, how much sexual desire did you experience?’ Responses were on a 1–7 scale (1 = ‘Not at all’; 4 = ‘A moderate amount’; 7 = ‘A lot’), and the items were highly intercorrelated with each other (Cronbach’s alpha = 0.96); responses to the three items were summed to form the composite. Two survey items were identified to assess courtship efforts. First, a single item inquired about overall mate attraction efforts on each day: ‘How much effort did you put into attracting a possible romantic and/or sexual partner yesterday?’ (same 1–7 scale as above). Second, because such efforts may be highly dependent on social exposure to potential partners, we also targeted a binary measure of such exposure: ‘Yesterday, did you have a direct social interaction with anyone you found attractive as a potential romantic and/or sexual partner, but who was not your romantic or sexual partner at the time?’ (Yes/No). Because survey items referred to ‘yesterday,’ responses were aligned with hormone concentrations from the previous day. Participants also recorded the time they woke up on each day, which was later compared with the recorded time of saliva collection to compute a difference between these times to control for circadian fluctuations in hormone concentrations (see §2d).

### (d) Hormone assays

The saliva samples were shipped, on dry ice, to the Hominoid Reproductive Ecology Laboratory at the University of New Mexico for testing. Testosterone was assayed in duplicate using enzyme-linked immunoassay (ELISA) kits (Salimetrics, USA). Cortisol was assayed with ELISA protocols (cortisol antibody, R4866) from the UC Davis Clinical Endocrinology Laboratory. For testosterone, the intra-assay coefficient of variation (CV) was 3%, and interassay CVs were 7.9% for high control and 12.5% for low control samples (*n* = 31); controls were within the manufacturer’s target range. For cortisol, the intra-assay CV was 5%, and interassay CVs were 12.5% for high control and 13.2% for low control (*n* = 65). Hormone concentrations more than three standard deviations from the population mean were excluded from further analyses. Inclusion of these values did not change any statistical conclusions—analyses including outliers are reported in electronic supplementary material.

Linear regression models were run to assess whether time elapsed between waking and the time of sample collection on any given day predicted testosterone and cortisol values. Time since waking measured in minutes was significantly and negatively associated with both testosterone (*β* = −0.237, *p* < 0.001) and cortisol (*β* = −0.213, *p* < 0.001) concentrations. To control for this effect, residuals from these models were saved and used in subsequent analyses in place of raw hormone values.

### (e) Statistical analyses

Multi-level regression models were employed to assess the within-subject relationships between same-day hormone concentrations and self-report survey measures. Hormone residuals extracted from the time of day analyses, along with the other predictor variables, were first grand-mean standardized and then subject-mean centred. Regression coefficients thus assess the effects of hormones fluctuations around each participant’s own mean, expressed in grand-mean standard deviation units. Because weekend samples lacked time-since-waking information, only weekday samples were used in our primary data analyses, but additional models that use all days without correcting for time of day appear as robustness checks in the supplementary online information. Model construction followed the approach outlined by Bates *et al*. [52], employing maximal models (including random intercept and slope terms) with uncorrelated random effects as our baseline models when convergence was possible, and removing upper-level random effects to aid convergence if necessary. Standardized regression coefficients (*β*) were estimated for our mixed-effects models, which reflect the expected s.d. change in outcome measures for a 1 s.d. change in the relevant predictor. The function used to calculate these coefficients can be found in electronic supplementary material, S5. In response to requests from reviewers, two level-2 covariates were added to the multi-level regression models that tested within-subject relationships between hormones and either desire or courtship efforts: relationship status and subject mean hormone concentrations. No zero-order relationships between daily hormone measures and the dependent variables were changed via the inclusion of these covariates.

We assessed our power to detect relationships between testosterone and desire or courtship effort using the ‘simr’ package in R [53]. Using the observed variances and final observation counts in our sample, the model generated simulated datasets with varying effect sizes for the relationships between testosterone and the desire or courtship effort dependent variables. For the day-to-day, within-person (level-1) associations, these simulations estimated greater than 95% power to detect small effect sizes (*β* = 0.10) and 80% power to detect effect sizes of 0.04 or larger. Power was much more limited for testing between-subjects (level-2) relationships between hormones and dependent variables, with 80% power to detect a large effect size (*β* = 0.60) (see electronic supplementary material, S4.1 and S4.2, for full model syntax and outputs.)

To address limitations in past research regarding the prediction and testing of time-lagged effects, we applied CT models to analyse within-subject, day-to-day lagged effects of testosterone and sexual desire on each other. Continuous-time models represent independent data points as continuous processes by fitting the data to a specified model using stochastic differential equations [47]. These allow for extraction of auto-effects, representing the predictive stability of variables over time (i.e. the within-subject effect of variable A on itself at later time points) and cross-effects, representing the predictive effect of one variable on another (i.e. the within-subject effect of variable A on variable B at later time points). We applied this method using the ctsemOMX R package [54]. Variables used in the CT models were person-mean centred and standardized but were otherwise the same as those used in the regression models. Beyond identifying the presence of significant lagged influences, CT models can be converted to discrete-time representations to examine these estimates with greater temporal detail. This allows one to extract the precise timing of effects, plotted as cross-regressive estimates, showing how variable relationships evolve and dissipate at different time interval lengths (e.g. variable A is most strongly correlated with variable B measured 2.5 days later, but weaker associations exist at shorter and longer time lags). For more details and examples, see [46,48]. The R markdown files specifying the analysis code for all analyses in this paper are available as electronic supplementary material, S5.

## 3. Results

### (a) Descriptives

A total of 1271 survey responses were recorded, and hormone measures were collected for 1143 of these. Hormone measures more than three standard deviations from the mean (and survey measures corresponding to that day; *n* = 14) were excluded from the main analyses. Descriptive statistics for all remaining data were as follows: testosterone (*M* = 142.15, s.d. = 52.47 pg ml^−1^, minimum = 34.41, maximum = 313.98), cortisol (*M* = 6.5, s.d. = 4.12 ng ml^−1^, minimum = 0.62, maximum = 22.55), courtship effort (*M* = 2.06, s.d. = 1.55, minimum = 1, maximum = 7) and sexual desire (*M* = 10.8, s.d. = 5.2, minimum = 3, maximum = 21).

As described in §2d, residuals of hormone measurements controlling for time since waking were used in our primary analysis. Because waking time was not collected for weekend samples, we were only able to use a subset of 759 responses for our primary analyses. Descriptive statistics for key variables in this weekday subset were extremely similar to those in the full dataset: testosterone (*M* = 145.89, s.d. = 48.15 pg ml^−1^, minimum = 48.56, maximum = 313.98), cortisol (*M* = 6.73, s.d. = 3.97 pg ml^−1^, minimum = 0.95, maximum = 22.55), courtship effort (*M* = 2.03, s.d. = 1.53, minimum = 1, maximum = 7) and sexual desire (*M* = 10.68, s.d. = 5.14, minimum = 3, maximum = 21). Testosterone and cortisol concentrations were significantly higher in single versus partnered men, and there were trends toward higher sexual desire among partnered men but higher courtship effort among single men (see electronic supplementary material, table S1).

### (b) Testosterone and sexual desire

**Figure 1.**
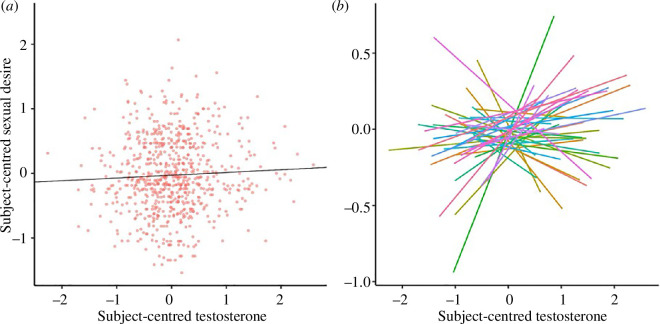
Observed relationships between subject-centred testosterone and sexual desire. Values for both variables were first grand-mean standardized and then subject-centred. (*a*) All subject-centred testosterone measures and associated (same-day) sexual desire values, with a regression line representing the overall relationship between the two variables (slope = 0.027). (*b*) Participant-level associations between testosterone and sexual desire, with each line representing an individual participant.

Table 2 presents tests of associations between testosterone and sexual desire with relationship status also entered as a covariate. Subject mean testosterone was not significantly associated with mean sexual desire. There was also no evidence for a significant association between daily testosterone measures and daily self-reports of sexual desire. Figure 1 depicts the clear absence of any zero-order, within-subject relationship between these variables: panel (*a*) shows all daily measures of subject-centred sexual desire against subject-centred testosterone, and panel (*b*) shows the relationship between these variables grouped by participant, with each line reflecting subject-level regression estimates (extracted from ordinary least squares regressions run independently for each participant’s data). Although the data in figure 1*b* suggest the possibility that a subset of participants may have significant associations between daily testosterone and desire, simulations presented in electronic supplementary material show that data drawn from random distributions can produce very similar patterns (see electronic supplementary material, S1.3). Follow-up analyses in electronic supplementary material also demonstrate that the non-significant associations between within-subject shifts in testosterone and desire were found in both single and partnered participants (see electronic supplementary material, S1.4).

**Table 2.** Mixed-regression model predicting sexual desire from same-day testosterone, mean testosterone and relationship status. As subject-level random slopes for daily testosterone did not converge, they were not included in the model.

fixed effects						
item	estimate	std. error	d.f.	*t*	*p-*value	95% CI
(intercept)	−0.107	0.161	37.910	−0.666	0.509	[−0.417, 0.203]
same-day testosterone	0.041	0.034	672.211	1.222	0.222	[−0.025, 0.107]
mean testosterone	−0.298	0.185	37.929	−1.610	0.116	[−0.656, 0.06]
relationship status	0.224	0.296	37.920	0.756	0.454	[−0.349, 0.797]

random effects						
item	variance	std. error	Wald’s *Z*	*p*‐value	95% CI	
(intercept)	0.617	0.146	4.219	<0.001	[0.33, 0.904]	

**Figure 2.**
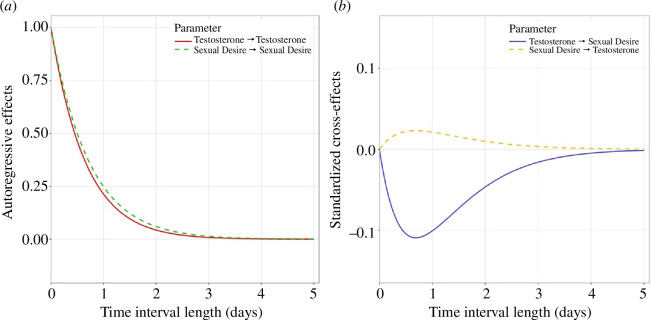
Discrete-time estimates of (*a*) autoregressive and (*b*) cross-regressive effects for testosterone and sexual desire. The *y*-axis represents the standardized estimate predicted given a 1 s.d. increase in the predictor variable.

The CT approach enabled us to test for the presence of time-lagged effects between within-subject fluctuations in testosterone and sexual desire. We first identified significant auto-effects for both testosterone and sexual desire. These auto-effects, as shown in figure 2*a* and detailed in electronic supplementary material, table S2, indicate that each variable consistently predicted itself at later time points. Specifically, measurements of either testosterone or sexual desire on one day correlated with measurements taken on subsequent days. The predictive stability over time for both testosterone and sexual desire was remarkably similar, with their lines almost entirely overlapping in the discrete-time plot (figure 2*a*). Both auto-effects appear to decrease substantially by the time 2 days have passed, indicating that a given hormone measure was significantly predictive of concentrations over the next 2 days, but not much further.

Cross-effects in CT models assess whether values of one variable predict those of another variable at future time points. While there was no significant cross-effect of sexual desire on testosterone levels in our data, there was an unexpected negative cross-effect of testosterone on sexual desire (electronic supplementary material, table S2). Specifically, higher levels of testosterone were associated with lower future sexual desire. This finding, contrary to our expectations, is displayed in the discrete-time plot of results (figure 2*b*), where cross-regressive estimates are standardized for interpretation. Shown by the testosterone-desire line (solid blue line), a 1 s.d. increase in testosterone predicted a significant decrease in sexual desire, peaking (estimate = −0.12) at around 0.7 days later. Conversely, a 1 s.d. increase in sexual desire predicted a small, non-significant rise in testosterone (estimate = 0.02) over a similar time interval. Cross-effects models run separately for single and partnered participants demonstrated that this negative cross-effect of testosterone on sexual desire was found among men in relationships but not among single men (see electronic supplementary material, tables S3 and S4); no positive lagged associations between testosterone and desire were found for either subgroup.

### (c) Testosterone and courtship effort

**Figure 3.**
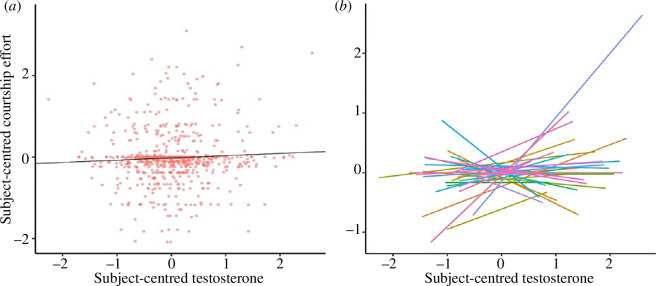
Observed relationships between subject-centred testosterone and courtship effort. Values for both variables were first grand-mean standardized and then subject-centred. (*a*) All subject-centred testosterone measures and associated (same-day) courtship effort values, with a regression line representing the overall relationship between the two variables (slope = 0.055). (*b*) Participant-level associations between testosterone and courtship effort, with each line representing an individual participant.

Table 3 presents tests of associations between testosterone and courtship effort with relationship status also entered as a covariate. Contrary to our prediction, there was no significant association between daily testosterone concentrations and daily self-reports of courtship effort. The absence of a significant relationship can be seen in figure 3, which displays subject-centred courtship effort versus that day’s subject-centred testosterone. As in figure 1, panel (*a*) displays all observations, whereas panel (*b*) displays regression lines for each participant. As shown in table 3, the random effect for the same-day testosterone association with courtship effort was not statistically significant; daily associations were also not significant when relationships between testosterone and courtship effort were tested separately for paired and single participants (see electronic supplementary material, S1.5). The CT modelling of testosterone and courtship effort produced significant auto-effects for both variables but no significant cross-effects (see electronic supplementary material, table S5).

**Table 3.** Mixed-regression model predicting courtship effort from level-1 testosterone, level-2 (mean) testosterone and relationship status.

fixed effects						
item	estimate	std. error	d.f.	*t*	*p*‐value	95% CI
(intercept)	0.084	0.150	38.022	0.559	0.579	[−0.206, 0.374]
same-day testosterone	0.062	0.049	27.794	1.269	0.215	[−0.035, 0.159]
mean testosterone	0.150	0.173	38.048	0.866	0.392	[−0.185, 0.484]
relationship status	−0.291	0.277	38.039	−1.051	0.300	[−0.826, 0.244]

random effects						
item	estimate	std. error	Wald’s *Z*	*p*‐value	95% CI	
(intercept)	1.283	0.308	4.164	<0.001	[0.68, 1.89]	
same-day testosterone	0.074	0.048	1.524	0.127	[0.00, 0.17]	

We conducted a set of exploratory analyses to further probe the possible relationship between testosterone and courtship effort. Mate attraction efforts may in part depend on interactions with potential mates, and such interactions may be much more frequent among single men. For daily self-reports of direct social interactions with new potential mates (DSI), we did in fact observe higher frequencies among single men: the average number of weekday occurrences across the month of data collection was 5.81 in single men and 2.14 in partnered men, with 7 partnered men having reported zero such interactions. A binary logistic mixed regression model confirmed a much greater probability of DSI among single versus partnered men: odds ratio = 7.03, *p* = 0.004, 95% CI = [1.85, 26.6]. Given the rarity of DSI occurrences in partnered men, and the expectation that mate attraction efforts are more important for single men, we tested whether DSI moderated the relationship between testosterone and courtship efforts specifically in single participants.

**Figure 4.**
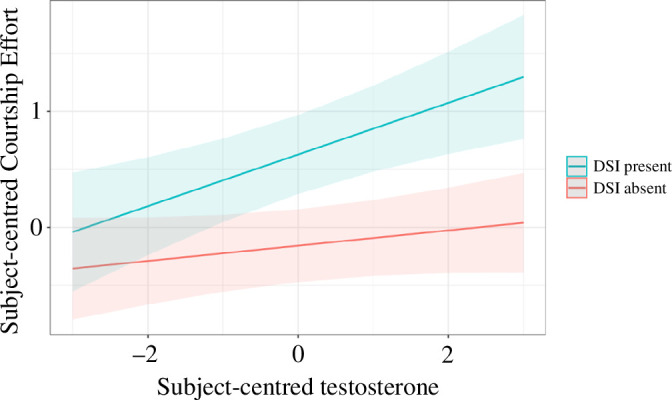
Courtship effort as a function of testosterone by DSI interaction for single men. The figure displays the estimated interaction effect of testosterone and DSI from our mixed-effects model predicting courtship effort (table 4). Shaded areas indicate 95% confidence intervals. DSI denotes a direct social interaction with a new potential mate on the survey response day.

Table 4 presents results of a linear mixed regression model that tested main effects of daily testosterone and DSI occurrence, and also the DSI by testosterone interaction, as predictors of mate attraction efforts. For these analyses, DSI was contrast-coded as −1 on days when such an interaction did not occur, and 1 when one did. There were significant positive effects for both DSI and daily testosterone concentrations, as well as a marginally significant interaction between these variables. Figure 4 displays the model predictions for the relationship between testosterone and courtship effort separately for days with and without DSI. A follow-up mixed-effects model using only days when DSI occurred showed a significant positive effect of testosterone on courtship effort (*β* = 0.159, d.f. = 138, *p* = 0.007, 95% CI = [0.045, 0.273]), which was absent on days when DSI did not occur (*β* = −0.039, d.f. = 4.9, *p* = 0.230, 95% CI = [−0.335, 0.257]). There was a trend towards testosterone concentrations being lower on DSI days among single men (*β* = −0.076, d.f. = 461, *p* = 0.054, 95% CI = [−0.153, 0.001]). Testosterone on DSI days was not a significant predictor of courtship effort for men in relationships (*β* = 0.087, d.f. = 27, *p* = 0.577, 95% CI = [−0.217, 0.391]).

**Table 4.** Mixed-regression model predicting courtship effort from testosterone, DSI (direct social interaction with a new potential mate on the survey response day) and their interaction in single men. As subject-level random slopes for daily testosterone did not converge, they were not included in the model.

fixed effects						
item	estimate	std. error	d.f.	*t*	*p*-value	95% CI
(intercept)	0.229	0.151	24.632	1.522	0.141	[−0.065, 0.524]
mean testosterone	0.029	0.190	23.692	0.153	0.879	[−0.34, 0.402]
same-day testosterone	0.144	0.042	432.854	3.416	<0.001	[0.062, 0.227]
DSI	0.393	0.085	23.906	4.633	<0.001	[0.222, 0.56]
same-day testosterone × DSI	0.078	0.042	431.458	1.854	0.064	[−0.004, 0.162]

random effects					
item	estimate	std. error	Wald’s *Z*	*p*‐value	95% CI
(intercept)	0.503	0.155	3.233	0.001	[0.198, 0.807]
DSI	0.138	0.052	2.686	0.007	[0.037, 0.24]

### (d) Moderation of testosterone effects by cortisol

To test the predictions of the dual hormone hypothesis, which proposes that cortisol levels moderate testosterone effects, we repeated the mixed-effect models with the inclusion of cortisol measures and a cortisol by testosterone interaction term. The model predicting sexual desire did not converge with random slopes for the interaction term or the testosterone term, so the model was run with random slopes for cortisol only; the results indicated no significant predictors of sexual desire (all *p* > 0.05). The mixed-effects model predicting courtship effort from testosterone and cortisol converged successfully at baseline, and likewise revealed no significant fixed effects (all *p* > 0.05). Full reports of these analyses, as well as models testing effects of cortisol without controlling for effects of testosterone (for which there were no significant effects), are presented in electronic supplementary material, S1.6.

### (e) Robustness checks

Robustness checks were run on the linear mixed regression models predicting same-day sexual desire and courtship effort from testosterone measures, using raw testosterone rather than residuals from time-since-waking models. We also tested whether statistical conclusions were affected by the inclusion or exclusion of outliers, as well as the inclusion or exclusion of weekend samples (which were excluded from primary analyses). In none of the robustness checks was testosterone significantly associated with sexual desire. The significant relationships between testosterone and courtship effort reported in §3c (e.g. on DSI present days) remained significant for all robustness checks except for those that included weekend samples (see electronic supplementary material, S2).

## 4. Discussion

### (a) The relationship between testosterone and sexual desire

This study provides direct, naturalistic evidence that day-to-day fluctuations in men’s sexual desire are not significantly associated with daily fluctuations in their testosterone concentrations. In other words, a man experiencing higher-than-usual testosterone concentrations on a given day does not typically exhibit higher-than-usual sexual desire on that same day (see figure 1). This finding is consistent with past within-subject studies that sampled on weekly [27] or monthly [28,29] time scales, with the present study adding important evidence via its analysis of a large volume of daily samples. We also found no evidence that men with higher mean testosterone across the study experienced higher mean sexual desire, which is also consistent with results from prior studies (see table 1).

Our study appears to be the first to have modelled continuous, time-lagged relationships between testosterone and sexual desire. Testosterone could affect desire at time delays that result from the genomic effects of the hormone [45], and a significant advantage of daily sampling is that it allows tests of whether, for instance, higher testosterone on one day predicts higher desire on subsequent days. The CT model that we employed allowed examination of relationships between the two variables across all possible time delays within a single model. Using this approach, we found a small, negative association between testosterone and future sexual desire (see ‘cross-effects’ in electronic supplementary material, table S2; figure 2*b*). This result was unexpected and warrants tests of its replication, but the negative association may provide additional evidence against the idea that men’s sexual desire positively tracks fluctuations in their testosterone concentrations within the normal range.

Studies involving testosterone administration or suppression have typically supported a threshold effect, in which sexual desire is only suppressed when testosterone concentrations are abnormally low, and raising testosterone beyond this hypogonadal threshold has no effect (reviewed in [55]). The results of our study are consistent with this hypothesized threshold effect; since the testosterone concentrations of our participants fell within the normal range, a positive relationship with sexual desire was not expected, nor was one observed. Our findings thus corroborate results from testosterone manipulation studies, and in conjunction with the few other studies that have measured within-subject correlations, provide naturalistic data that converge on the conclusion that testosterone variability in the normal range does not predict variability in men’s self-reported sexual desire.

### (b) The relationship between testosterone and courtship effort

Though testosterone does not appear to positively regulate fluctuations in sexual desire, it may instead motivate mating effort through other means, such as by promoting courtship effort. Our results failed to support positive relationships between testosterone and self-reported efforts at mate attraction when considering all data points (see figure 3 and table 3). Exploratory follow-up analyses, however, did provide evidence for a positive association between courtship effort and testosterone among single men on days when the participants reported a direct social interaction with potential mates (DSI). In other words, among days with DSI, self-reports of courtship effort were higher when testosterone was higher. This effect can be seen visually in figure 4 (regression line for DSI present). Intuitively, opportunities for courtship effort may simply be lower without DSI, thus explaining the non-significant testosterone-courtship effort relationship on DSI absent days, and when considering all days. We stress that a testosterone-courtship effort relationship restricted to single men on DSI present days was not predicted *a priori* and emerged in exploratory analyses, which highlights the importance of testing replication of this effect in an independent sample.

The causal relationship between testosterone and courtship effort on DSI present days is ambiguous from our data. Saliva samples were collected in the early afternoon, meaning that encounters with potential mates may have occurred before or after testosterone was measured. This presents two alternatives: higher testosterone may have driven higher courtship effort in encounters later in the day, or more effortful courtship interactions earlier in the day may have triggered reactive increases in testosterone concentrations (e.g. [39,56]). Intuitively, one expects that most interactions with potential mates would occur later in the day, which if true, would be consistent with higher testosterone on a given day having caused greater mate attraction efforts. This could be tested more systematically by having men engage in social interactions with potential mates across multiple days and then assessing whether measured courtship efforts were greater on days with higher baseline testosterone. Despite the causal ambiguity in our data, our findings provide preliminary support for a day-to-day relationship between men’s testosterone and their courtship efforts.

Our results are generally consistent with theoretical models that propose that testosterone mediates life history transitions between mate competition and partnering/parenting in human males, as in other species [34,35,37,38]. These models essentially posit that elevated testosterone functions to promote mate competition efforts in single men, but that relationship entry as an effect of those efforts may in turn lead to lower testosterone production with a concomitant shift in effort away from courtship and toward investment in pair bonds and paternal provisioning. Single men had higher mean testosterone than partnered men in our sample—replicating past findings (see [36] for meta-analytic review)—and also engaged in more frequent interactions with potential mates. In addition, for single men only, mate attraction efforts on days with DSI were higher when testosterone was higher, which provides preliminary but original evidence that testosterone may in fact promote mate attraction efforts among unpartnered men.

### (c) Limitations

Our sample comprised undergraduate men in an industrialized country and it is uncertain whether our findings would generalize to other age groups or socioecological environments. Although we found that testosterone and sexual desire were not significantly, positively associated overall (a pattern that was found in both single and partnered men), it is possible that a positive relationship might be found in other subgroups of men. Our measures of sexual desire did not differentiate between dyadic and solitary desire, which have been differentially associated with testosterone in some samples of women [19], and future research might assess these subtypes of desire. It is possible that desire and testosterone are positively associated at shorter time scales than what we measured. Testosterone increases have been demonstrated within about 15 min after exposure to visual erotica [57], with such exposures likely to also induce immediate increases in sexual desire. Nonetheless, our current findings suggest that any such short-term linkages between testosterone and desire are not sufficient to produce positive correlations between these variables at the day-to-day time scale. Finally, the exploratory and correlational nature of our results showing positive associations between testosterone and courtship effort suggest the importance of future research that can further test the role of testosterone in men’s mate attraction efforts.

### (d) Conclusion

Our findings provide naturalistic evidence that day-to-day testosterone fluctuations in the eugonadal range do not positively predict fluctuations in men’s sexual desire, either concurrently or at time-lags. These findings corroborate prior research in suggesting that men’s sexual desire requires only a threshold amount of baseline testosterone above which testosterone changes do not reliably affect desire. That conclusion in turn argues against the usefulness of testosterone prescriptions for treating low sexual desire among men who have testosterone concentrations within the normal range. Exploratory analyses produced evidence that testosterone fluctuations in the normal range may positively predict day-to-day changes in men’s mate attraction efforts among single men, especially given social interactions with potential mates. Future research could test replication of that pattern and seek to disentangle the possible causal pathways through which associations between testosterone and courtship effort may arise.

## Data Availability

All data and analysis code are available at Open Science Framework [58]. Supplementary material is available online [59].
